# Functions of nonsuicidal self-injurious behavior in Russian patients with suicidal ideation

**DOI:** 10.3389/fpubh.2023.1270944

**Published:** 2023-11-07

**Authors:** Mikhail Zinchuk, Georgii Kustov, Sofya Popova, Ilya Mishin, Nadezhda Voinova, Anna Gersamija, Alexander Yakovlev, Alla Guekht

**Affiliations:** ^1^Moscow Research and Clinical Center for Neuropsychiatry, Moscow, Russia; ^2^Institute of Higher Nervous Activity and Neurophysiology, Russian Academy of Sciences, Moscow, Russia; ^3^Pirogov Russian National Research Medical University, Moscow, Russia

**Keywords:** nonsuicidal self-injury, Inventory of Statements About Self-Injury, confirmatory factor analysis, validation, non-psychotic mental disorders

## Abstract

**Introduction:**

Nonsuicidal self-injurious behavior (NSSI) is an important risk factor for future suicide attempts. Previous research has identified a number of motivations for engaging in NSSI. The aim of the present study was to translate the Inventory of Statements About Self-Injury (ISAS) into Russian and then to evaluate its psychometric properties in a sample of patients with non-psychotic mental disorders and suicidal ideation (SI). Other aims were to determine the prevalence of specific NSSI functions in this population and to assess the relationship between different NSSI functions and clinical and psychological parameters.

**Participants and methods:**

The study was conducted at the largest center for non-psychotic mental disorders in Moscow. All admitted patients with both NSSI and SI completed the Russian version of the ISAS-II, underwent the Self-Injurious Thoughts and Behaviors Interview, and completed the Personality Inventory for DSM-5 and ICD-11 Brief Form Plus-Modified, the Beck Depression Inventory, and the State–Trait Anxiety Inventory.

**Results:**

A total of 614 patients were included in the study. 543 (88.4%) patients were assigned female at birth with a mean age of 24.86 (7.86) years. Factor analysis supported a two-factor structure (Intrapersonal and Interpersonal) of the Russian version of the ISAS-II, but in contrast to the original study, the “Marking distress” function loaded more strongly on the Interpersonal factor. In people with non-psychotic mental disorders and SI, Interpersonal functions of NSSI are associated with more severe depressive symptoms (*r* = 0.34), 12 months history of NSSI (*r* = 0.30), higher number of NSSI methods (*r* = 0.41), likelihood of future NSSI (*r* = 0.35) and psychoticism (*r* = 0.32).

**Conclusion:**

The Russian version of the ISAS-II is a valid and reliable instrument for assessing NSSI functions in a population at high risk for suicide attempts. Interpersonal functions are associated with a number of unpleasant clinical and psychological features.

## 1. Introduction

Suicide remains one of the leading causes of preventable death, particularly among young adults (1). The death rate from suicide has not shown a significant decline in recent decades (2), comparable to that observed for many other causes of premature death (3, 4). Despite the many studies carried out each year in this area, the problem remains unresolved and it is appropriate to continue the search for modified predictors of suicide.

Recent studies have identified nonsuicidal self-injury (NSSI) as a highly significant risk factor for future fatal and non-fatal suicide attempts (5–7). People with mental disorders who also have suicidal ideation and NSSI are clearly a group at highest risk of attempting suicide. At the same time NSSI is highly prevalent both in the general population (8) and particularly among people with mental disorders (9, 10) and only a minority of them attempt suicide. Therefore, research into the characteristics of NSSI that distinguish those at high risk of suicide is warranted. Over the last decade, the number of studies on NSSI has increased, but the data obtained vary considerably depending on the methods used by the authors (11, 12). A set of instruments that are freely accessible, available in many languages and validated in different countries is a prerequisite for conducting comprehensive comparative studies on NSSI and suicidal behavior.

To date, none of the self-report instruments used by researchers worldwide to assess parameters associated with NSSI (13) has been validated in the Russian language. The lack of a tool to assess the functioning of NSSI hinders progress in the development of appropriate suicide prevention programs in Russia. It’s worth noting that suicide remains a major public health problem in the Russian Federation as the country’s suicide mortality rate has exceeded the European average for several decades (14). These data contrast with the relatively small number of published papers on suicidal behavior (15), and very few of these have considered NSSI as a potential risk factor for suicidality (16–18). Among the many reasons for ignoring this very important issue, the lack of validated Russian-language instruments for NSSI studies may be one of the most important.

Another benefit of validating the instrument for assessing NSSI functions is the development of more precise treatment strategies for individuals with specific NSSI motives. For example, programs aimed at increasing emotional tolerance (e.g., Dialectical Behavior Therapy) may be recommended for patients for whom affect regulation is the dominant motive for NSSI, whereas programs aimed at improving communication skills and family therapy may be recommended for those for whom distress communication is the primary function (19–21).

The Inventory of Statements About Self-Injury (ISAS) (22, 23) is a freely available tool that provides information about NSSI parameters such as type of NSSI, age of onset, date of last NSSI, experience of physical pain, willingness to stop self-harm (ISAS-I), and key functions of NSSI (ISAS-II). The instrument has been validated in many languages including Serbian (24), Spanish (25), Hungarian (26), Persian (27), Korean (28), Turkish (29), Urdu (30), and Swedish (31), and is currently in active use worldwide. Most of the translated versions of this instrument showed good internal consistency and the 2-factor structure of the ISAS-II (intrapersonal and interpersonal factors), which is consistent with the results of the original study. For example, in a Turkish study factor analysis of the functions scale confirmed the good fit of the original 2-dimensional model (RMSEA = 0.08 (0.07–0.09); CFI = 0.97; NFI = 0.97) (29). Cronbach’s alpha was 0.92 for the Korean version of the ISAS-II, indicating excellent internal consistency reliability (28). The test–retest reliability of the instrument was also found to be good (32).

Previous studies that using the ISAS-II have found that certain NSSI motives are more strongly associated with various adverse outcomes than others. For example, Reinhardt et al. (33) found that intrapersonal, but not interpersonal NSSI functions were associated with indicators of NSSI severity (current and recurrent NSSI, versatility of methods). In addition, a number of psychopathological features (co-occurring mental disorders, presence of a mood disorder, internalizing symptoms of mental illness, and self-critical rumination) were associated with engagement in NSSI for intrapersonal reasons. Thus, studies using the ISAS to assess the functions of NSSI may provide insight into the place of different subtypes of NSSI within the psychopathological taxonomy.

However, even though the ISAS is not a diagnostic tool and does not require a cut-off point, the use of simply translated versions of the instrument without studying its psychometric properties (31, 34) should not be considered good practice. For example, even for instruments with fewer questions, inconsistencies in the factor structure between the original and Russian-language versions have been reported (35, 36). Moreover, in the Iranian study (37), the EFA showed a single-factor solution that provided an adequate fit in the subsequent confirmatory factor analysis (CFA). In addition, a Japanese study (38) reported a three-factor structure of the instrument: “Distress coping functions,” “Interpersonal influence functions,” and “Identity maintenance functions.” Also, the study conducted in a Mexican student population (39) found 7 interpretable factors (“Self-regulation,” “Revenge,” “Sensation seeking/toughness,” “Avoiding suicide,” “Marking distress,” “Self-determination and numbness”), 5 more than the original version of the ISAS-II. These data support the need for a psychometric study of the Russian version of the ISAS-II prior to its use.

The primary aim of the study was to translate the ISAS-II into Russian language and then to examine its psychometric properties in a sample of Russian-speaking patients with non-psychotic mental disorders and suicidal ideation. It seems crucial to understand the characteristics of the instrument in this special population, which is characterized by an enormously high risk of suicide attempts.

The next aims of the study were to obtain data on the prevalence of various NSSI functions in a consecutive sample of Russian patients with non-psychotic mental disorders and suicidal ideation, and to assess the relationship between various NSSI functions and clinical (diagnosis, depression and anxiety levels, self-harm thoughts and behaviors) and psychological (personality traits) characteristics.

Patients with primary psychotic disorders were not included in this study because we believe that a separate study of NSSI functions in these patients is warranted. This is because many key parameters for understanding these patients are not applicable to people with non-psychotic mental disorders (e.g., duration of untreated psychotic symptoms, impaired insight, and negative symptoms). Each of these parameters could potentially affect the motivation for NSSI, and we decided not to include people with primary psychotic disorders in the study.

## 2. Participants and methods

### 2.1. Procedure

The study was conducted at the Department of Suicide Research and Prevention at the Moscow Research and Clinical Center for Neuropsychiatry. The Center specializes in the treatment of patients with non-psychotic mental disorders. The study cohort is represented by patients with SI and NSSI aged >18 years and older, identified from a consecutive cohort of patients with non-psychotic mental disorders and suicidal ideation. Patients with primary psychotic disorders, current substance use disorders, cognitive deficits below the level of comprehension on self-report scales and interviewer questions were excluded from the study.

All patients are screened on admission for lifetime SI, suicide attempts (SA), and NSSI. The screening includes the first items in the relevant sections of the Self-Injurious Thoughts and Behaviors Interview (SITBI) – “Have you ever had thoughts of killing yourself?” “Have you ever actually made a plan to kill yourself?” and “Have you ever actually engaged in NSSI?” All patients are then seen by an experienced psychiatrist to confirm the diagnosis of a non-psychotic mental disorder according to ICD-10 criteria.

All eligible patients then completed the study questionnaires and were interviewed by the investigator to collect basic socio-demographic information and data on self-injurious thoughts and behaviors. The first patient was enrolled in January 2018 and the last in December 2019.

### 2.2. Measures

The Inventory of Statements about Self-Injury-II (ISAS-II) consists of 39 items with responses rated on a 3-point Likert scale from 0 (not relevant) to 3 (very relevant). The list of items begins with an opening statement: “When I self-harm, I am…” According to the authors of the original study, the ISAS-II has two higher-order functions (Interpersonal and Intrapersonal) and 13 lower-order facets. The Intrapersonal function in the original ISAS-II version consists of motives for NSSI such as “Affect regulation” (… releasing emotional pressure that has built up inside of me), “Anti-dissociation/feeling-generation” (… causing pain so I will stop feeling numb), “Anti-suicide” (… avoiding the impulse to attempt suicide), “Marking distress” (… creating a physical sign that I feel awful), “Self-punishment” (… punishing myself) and the Interpersonal function includes “Autonomy” (… demonstrating that I do not need to rely on others for help), “Interpersonal boundaries” (… demonstrating that I am separate from other people), “Interpersonal influence” (… letting others know the extent of my emotional pain), “Peer bonding” (… bonding with peers), “Revenge” (… getting back at someone), “Self-care” (… creating a physical injury that is easier to care for than my emotional distress), “Sensation seeking” (… doing something to generate excitement or exhilaration) and “Toughness” (… seeing if I can stand the pain). The Cronbach’s alpha coefficient of the original ISAS-II version indicated good internal consistency for the interpersonal (0.88) and intrapersonal (0.80) factors (23).

With the permission of the author of the instrument (prof. ED Klonsky), the Russian version of the ISAS-II (Appendix 1) was developed using the back-translation method, which is recommended as a first step in cross-cultural adaptation of instruments. The original version of the ISAS-II was translated from Russian by two native Russian psychiatrists, both of whom were fluent in English. The Russian version of the tool was then back-translated into English by another bilingual translator on the research team to confirm that the translation was consistent with the wording of the original scale. Finally, the Russian version was compared with the original version by an English-speaking consensus committee of clinicians. If there were discrepancies between the two versions, individual items underwent additional rounds of back-translation until they were deemed satisfactory. The developed version of the tool was tested on 16 patients with NSSI (8 females). In their feedback, none of the participants reported any difficulties in understanding the instructions for the tool or the meaning of the items. This version of the ISAS-II was later used in our study.

The Self-Injurious Thoughts and Behaviors Interview (SITBI) is a structured clinical diagnostic interview consisting of the following blocks: suicidal thoughts, suicide plans, suicide attempts, suicidal gestures, non-suicidal self-injurious thoughts and non-suicidal self-injurious behaviors (40). Each block contains up to 30 questions on prevalence, frequency, and contextual factors. The Russian version of the SITBI is mainly used for scientific purposes (17, 41), but in some clinical centers it is also used in routine clinical practice. For the purposes of this study, we used the SITBI questions on prevalence and age of onset of SI, SA, and NSSI, as well as items on methods of self-injury and questions assessing descriptive and contextual factors of NSSI.

The Personality Inventory for DSM-5 and ICD-11 Brief Form Plus-Modified (PID5BF + M) is part of the DSM-5 family of personality trait questionnaires (42, 43). The PID5BF + M was proposed by Bach et al. (44) and consists of 36 questions rated on a 4-point Likert scale from 0 (very untrue or often untrue) to 3 (very true or often true). The questionnaire assesses 6 domains, including 3 facets, each consisting of two questions: Negative Affectivity (emotional lability, anxiousness, and separation insecurity), Detachment (withdrawal, anhedonia, intimacy avoidance), Antagonism/Dissociality (manipulativeness, deceitfulness, grandiosity), Disinhibition (impulsivity, irresponsibility, distractibility), Anankastia (rigidity, perfectionism, and orderliness), and Psychoticism (unusual beliefs, perceptual dysregulation, eccentricity). The instrument showed good internal consistency (McDonald’s omega coefficients ranged from 0.83 to 0.90) and its factor structure was fully consistent with the results of the study of the original version of the questionnaire (45).

The Beck’s Depression Inventory (BDI) was developed to assess the severity of depressive symptoms (46). It consists of 21 items, each rated on a 4-point Likert scale from 0 to 3. The psychometric properties of the Russian version of the BDI have been previously evaluated, with Cronbach’s alpha coefficients of 0.86 (47).

The State–Trait Anxiety Inventory (STAI) consists of two parts assessing state (STAI-S) and trait (STAI-T) anxiety (48). Each part contains 20 items and is scored on a 4-point Likert scale ranging from 1 (not at all) to 4 (very much so). The questionnaire has previously been validated in Russian by Khanin (49), with Cronbach’s alpha of 0.89 and 0.85 for the state and trait anxiety modules, respectively (50).

### 2.3. Statistical analysis

Categorical variables are presented as frequencies (%) and continuous variables as arithmetic means (standard deviation).

Factor structure was assessed using exploratory factor analysis (EFA) with standard geomin (oblique) rotation and the weighted least square mean and variance adjusted (WLSMV) estimator. WLSMV is a robust estimator suitable for responses with four or fewer response categories and can be corrected for non-normality in the data set (51–53). Kaiser-Meyer-Olkin (KMO) and Bartlett’s sphericity tests were used to assess the suitability of the data for EFA.

The internal consistency of the questionnaire was assessed using McDonald’s omega scores (ω) (53). Average inter-item correlations of the NSSI functions were also calculated to assess item homogeneity (54).

Correlational analysis was used to assess the relationship between NSSI functions and sociodemographic and clinical variables. Correlations between inter/intrapersonal functions and binary variables (age, mental disorder diagnoses, lifetime and 12 months suicide attempts, and 12 months NSSI and NSSI medical treatment) were assessed using biserial correlations. Polyserial correlations were used to examine associations between NSSI functions and ordinal variables (SITBI items 153–162, 165–169) and Pearson’s correlations for continuous variables.

Calculations were performed using Mplus v7.0, Jamovi v2.3.17.0 and BlueSky Statistics v10.3.0.

## 3. Results

### 3.1. Sample characteristics

Of the 3,644 patients screened, 655 (18.0%) were positive for lifetime SI and NSSI. Forty-one patients refused to participate in the study or completed the ISAS-II incorrectly. Thus, 614 patients were included in the final study calculation, of which 14 refused to undergo the SITBI but did not object to the use of their completed questionnaire data in the calculation. Data from these patients were therefore excluded from the analysis of the relationship between NSSI function and the SITBI variables.

Clinical and demographic variables are presented in Table 1. The majority of patients were assigned female at birth (*N* = 543; 88.4%) with a mean age of 24.86 (SD 7.86; range 18–72) years. Fifty-one patients (8.3%) had an alternative gender identity (those with a non-binary gender identity (e.g., gender fluid, agender, and bigender) and those receiving gender affirming care).

**Table 1:** Sociodemographic and clinical characteristics of the sample.

Parameter	Mean (SD)
**Age**	24.86 (7.86)
**Age of onset of suicidal ideation**	15.66 (6.45)
**Age at first suicide attempt**	18.1 (6.38)
**Age at onset of NSSI**	15.8 (6.4)
***N* of NSSI methods**	4.2 (2.0)
**BDI**	30.81 (10.25)
**STAI-S**	61.48 (10.05)
**STAI-T**	62.07 (9.93)
**PID5BF + M**	
Negative affectivity	3.67 (1.4)
Detachment	2.31 (1.2)
Antagonism	2.16 (1.3)
Disinhibition	3.05 (1.28)
Anankastia	2.34 (1.54)
Psychoticism	2.35 (1.51)

Parameter	*N* (%)
**Sex assigned at birth**	
Male	71 (11.6%)
Female	543 (88.4%)
**Gender**	
Male	68 (11.1%)
Female	495 (80.6%)
Alternative gender identity	51 (8.3%)
**Education level**	
Elementary and middle school	32 (5.2%)
High school	102 (16.6%)
Secondary vocational education	85 (13.8)
Unfinished higher education	225 (36.6%)
Completed higher education	170 (27.7%)
**Employment status**	
Employed	379 (45.4%)
Retired	16 (2.6%)
Unemployed	319 (52.0%)
**Marital status**	
Single	343 (52.9%)
Married	69 (11.2%)
In another type of relationship (not formally married)	213 (34.7%)
**Mental disorder diagnoses**	
Schizophrenia spectrum disorder	77 (12.5%)
Bipolar disorder	160 (26.1%)
Major depressive disorder	165 (26.9%)
Anxiety disorder	79 (12.9%)
Obsessive-compulsive disorder	8 (1.3%)
Eating disorder	17 (2.8%)
Personality disorder	162 (26.4%)
Multiple psychiatric diagnoses	55 (9.0%)
**Lifetime suicide attempts**	270 (44.7%)
**12-month suicide attempts**	134 (22.2%)
**NSSI in the past 12 months**	457 (75.7%)
**NSSI methods**	
Cutting or carving	457 (75.7%)
Hitting	371 (61.4%)
Pulling hair out	150 (24.8%)
Self-tattooing	50 (8.3%)
Picking a wound	276 (45.7%)
Burning skin	214 (35.4%)
Inserting objects under the nails or skin	70 (11.6%)
Biting	322 (53.3%)
Picking body areas	171 (28.3%)
Scraping skin	319 (52.8%)
Erasing skin	89 (14.7%)
Other	48 (7.9%)
**Medical attention**	72 (12.0%)

NSSI, nonsuicidal self-injury; SITBI, self-injurious thoughts and behaviors interview; BDI, Beck’s depression inventory; STAI-S, state–trait anxiety inventory-sate; STAI-T, state–trait anxiety inventory-trait; PID5BF + M, personality inventory for DSM-5 and ICD-11 brief form plus–modified.

The most common diagnoses were affective disorders [bipolar disorder – 160 (26.1%); depressive disorder – 165 (26.9%)] and personality disorders [162 (26.4%)]. Fifty-five (9.0%) patients were diagnosed with more than one mental disorder. The lifetime prevalence of suicide attempts was 44.7%.

The mean age of onset of NSSI was 15.8 (6.4) years. Approximately 75% had engaged in NSSI in the past 12 months. The most common methods of NSSI were cutting or carving (*N* = 457; 75.7%), hitting (*N* = 371; 61.4%), biting (*N* = 322; 53.3%), and scratching (*N* = 319; 52.8%). The mean number of NSSI methods was 4.2 (2.0), and 77 (12%) of patients had previously sought non-psychiatric medical help for the consequences of NSSI.

### 3.2. Factor structure of the questionnaire

The EFA yielded a two-factor solution that accounted for 38.4% of the total variance. Factor 1 had an eigenvalue of 10.1 and included Intrapersonal functions, and Factor 2 had an eigenvalue of 4.9 and included Interpersonal functions. The two factors had an intercorrelation of 0.22. As can be seen in Table 2, 34 of the 39 item loadings were consistent with the loadings reported by Klonsky and Glenn (23). Three items (11, 24, and 37) loaded on the Interpersonal rather than the Intrapersonal factor, and item 17 loaded on the Intrapersonal rather than the Interpersonal factor. Item 7 was cross-loaded on two factors, but both factor loadings were low (0.25). In order to maintain the integrity of functions such as “Sensation Seeking” and “Self-care,” it was decided to place items 7, 17 in the original factors. Thus, Intrapersonal functions included functions such as “Affect regulation,” “Self-punishment,” “Anti-suicide” and “Anti-dissociation,” and interpersonal functions – “Marking distress,” “Self-care,” “Interpersonal influence,” “Toughness,” “Sensation-seeking,” “Interpersonal boundaries,” “Autonomy,” “Revenge” and “Peer bonding.”

**Table 2:** Factor loadings of the ISAS-II items.

No.	Item	Intrapersonal	Interpersonal	Original function	Original factor
1	Calming myself down	**0.47**	−0.10	Affect regulation	Intrapersonal
2	Creating a boundary between myself and others	0.22	**0.45**	Interpersonal boundaries	Social
3	Punishing myself	**0.61**	0.03	Self-punishment	Intrapersonal
4	Giving myself a way to care for myself (by attending to the wound)	0.06	**0.40**	Self-care	Social
5	Causing pain so I will stop feeling numb	**0.81**	−0.12	Anti-dissociation	Intrapersonal
6	Avoiding the impulse to attempt suicide	**0.75**	0.07	Anti-suicide	Intrapersonal
7	Doing something to generate excitement or exhilaration	0.25	**0.25**	Sensation-seeking	Social
8	Bonding with peers	−0.32	**0.71**	Peer-bonding	Social
9	Letting others know the extent of my emotional pain	−0.06	**0.73**	Interpersonal influence	Social
10	Seeing if I can stand the pain	0.27	**0.46**	Toughness	Social
11	Creating a physical sign that I feel awful	0.20	**0.59**	Marking distress	Intrapersonal
12	Getting back at someone	−0.16	**0.79**	Revenge	Social
13	Ensuring that I am self-sufficient	0.18	**0.64**	Autonomy	Social
14	Releasing emotional pressure that has built up inside of me	**0.60**	−0.11	Affect regulation	Intrapersonal
15	Demonstrating that I am separate from other people	0.03	**0.70**	Interpersonal boundaries	Social
16	Expressing anger towards myself for being worthless or stupid	**0.74**	0.06	Self-punishment	Intrapersonal
17	Creating a physical injury that is easier to care for than my emotional distress	**0.44**	0.17	Self-care	Social
18	Trying to feel something (as opposed to nothing) even if it is physical pain	**0.87**	−0.15	Anti-dissociation	Intrapersonal
19	Responding to suicidal thoughts without actually attempting suicide	**0.66**	0.13	Anti-suicide	Intrapersonal
20	Entertaining myself or others by doing something extreme	−0.02	**0.51**	Sensation-seeking	Social
21	Fitting in with others	−0.23	**0.65**	Peer-bonding	Social
22	Seeking care or help from others	0.00	**0.67**	Interpersonal influence	Social
23	Demonstrating I am tough or strong	0.01	**0.64**	Toughness	Social
24	Proving to myself that my emotional pain is real	0.36	**0.47**	Marking distress	Intrapersonal
25	Getting revenge against others	−0.18	**0.81**	Revenge	Social
26	Demonstrating that I do not need to rely on others for help	0.10	**0.60**	Autonomy	Social
27	Reducing anxiety, frustration, anger, or other overwhelming emotions	**0.62**	−0.10	Affect regulation	Intrapersonal
28	Establishing a barrier between myself and others	0.15	**0.64**	Interpersonal boundaries	Social
29	Reacting to feeling unhappy with myself or disgusted with myself	**0.67**	0.08	Self-punishment	Intrapersonal
30	Allowing myself to focus on treating the injury, which can be gratifying or satisfying	0.22	**0.35**	Self-care	Social
31	Making sure I am still alive when I do not feel rea	**0.67**	−0.01	Anti-dissociation	Intrapersonal
32	Putting a stop to suicidal thoughts	**0.73**	0.12	Anti-suicide	Intrapersonal
33	Pushing my limits in a manner akin to skydiving or other extreme activities	0.26	**0.40**	Sensation-seeking	Social
34	Creating a sign of friendship or kinship with friends or loved ones	−0.24	**0.63**	Peer-bonding	Social
35	Keeping a loved one from leaving or abandoning me	−0.02	**0.58**	Interpersonal influence	Social
36	Proving I can take the physical pain	0.29	**0.59**	Toughness	Social
37	Signifying the emotional distress, I’m experiencing	0.15	**0.68**	Marking distress	Intrapersonal
38	Trying to hurt someone close to me	−0.23	**0.69**	Revenge	Social
39	Establishing that I am autonomous/independent	0.10	**0.77**	Autonomy	Social

Primary loadings are in bold.

### 3.3. Internal consistency of the questionnaire

The McDonald’s omega coefficient for the ISAS-II was 0.85, indicating good internal consistency. The internal consistency coefficients for the functions of the NSSI are presented in Table 3. McDonald’s omega coefficients for Intrapersonal and Interpersonal functions were greater than 0.80. The internal consistency of most of the NSSI motives was acceptable (ω ≥ 0.70), except for the coefficients for “Self-Care,” “Sensation seeking,” “Interpersonal boundaries,” and “Peer bonding,” which ranged from 0.55 to 0.70, indicating less adequate reliability.

**Table 3:** Prevalence of NSSI functions and internal consistency and descriptive statistics for ISAS-II domains and facet scores.

	*N* (%)	Mean	SD	Skewness	Kurtosis	ω	Inter-item correlations
**Intrapersonal functions**		14.80	6.22	−0.11	−0.56	0.83	0.29
Affect regulation	590 (96.1%)	4.50	1.63	−1.12	0.61	0.71	0.44
Self-punishment	535 (87.1%)	3.65	2.09	−0.46	−1.11	0.83	0.62
Anti-suicide	466 (75.9%)	2.63	2.11	0.22	−1.26	0.83	0.61
Anti-dissociation	446 (72.6%)	2.55	2.15	0.27	−1.31	0.83	0.60
**Interpersonal functions**		7.90	6.57	1.22	2.10	0.85	0.18
Marking distress	484 (78.8%)	2.63	2.03	0.26	−1.13	0.75	0.49
Self-care	423 (68.9%)	1.47	1.35	0.89	0.68	0.55	0.26
Interpersonal influence	337 (54.9%)	1.35	1.59	1.00	−0.04	0.72	0.43
Toughness	330 (53.7%)	1.37	1.64	1.04	0.13	0.73	0.44
Sensation-seeking	260 (42.3%)	0.82	1.22	1.65	2.30	0.53	0.26
Interpersonal boundaries	185 (30.1%)	0.63	1.16	2.07	4.09	0.68	0.38
Autonomy	145 (23.6%)	0.52	1.16	2.70	7.48	0.75	0.49
Revenge	119 (19.4%)	0.39	1.00	3.36	12.40	0.78	0.53
Peer-bonding	73 (11.9%)	0.20	0.68	4.97	30.10	0.66	0.39

ISAS-II, the inventory of statements about self-injury; ω, McDonald’s omega coefficient.

The average inter-item correlation coefficient for the ISAS-II was 1.51. As shown in Table 3, the average inter-item correlations for the Interpersonal and Intrapersonal functions and most of the subfunctions were within the acceptable range of 0.15–0.50.

### 3.4. Prevalence of NSSI functions

As shown in Table 3, the most common functions of NSSI in non-psychotic patients with SI were “Affect regulation” (*N* = 590; 96.1%), “Self-punishment” (*N* = 535; 87.1%), “Marking distress” (*N* = 484; 78.8%), “Anti-suicide” (*N* = 466; 75.9%) and “Anti-dissociation” (*N* = 446; 72.6%). However, they rarely self-harmed for “Revenge” (*N* = 119; 19.4%) or “Peer bonding” (*N* = 73; 11.9%).

### 3.5. Factors related to the interpersonal and intrapersonal functions of NSSI

The results of the correlation analysis are presented in Table 4. A total of Intrapersonal self-injurious motives had significant positive weak correlations with and BDI score (*r* = 0.34) and negative significant weak correlations with age (*r* = −0.39). Significant weak positive correlations were found between Intrapersonal NSSI functions and psychoticism.

**Table 4:** Correlation analysis of NSSI functions with sociodemographic parameters, personality domains and clinical variables.

Parameter	Interpersonal functions	Intrapersonal functions
Age (#)	0.07	−0.39***
Male vs. female (@)	−0.04	0.27***
Schizophrenia spectrum disorder (@)	0.02	0.12
Bipolar disorder (@)	−0.05	0.05
Major depressive disorder (@)	−0.08	−0.08
Anxiety disorder (@)	0.00	−0.22***
Personality disorder (@)	0.12	013*
Multiple psychiatric diagnoses (@)	0.06	0.10
BDI score (#)	0.00	0.34***
STAI–S score (#)	0.07	0.26***
STAI-T score (#)	0.02	0.20***
**PID5BF + M**		
Negative affect (#)	0.13***	0.20***
Detachment (#)	0.04	0.16***
Antagonism (#)	0.25***	0.08
Disinhibition (#)	0.16***	0.21***
Anankastia (#)	0.18***	0.10**
Psychoticism (#)	0.20***	0.32***
**SITBI**		
(2) Age at SI onset (#)	−0.02	−0.18***
(84) Lifetime SA (@)	0.05	0.24***
(89) 12-month SA (@)	−0.08	0.22***
(85) SA age at onset (#)	0.12*	0.04
(88) *N* of SA (#)	0.05	0.08
(147) 12-month NSSI (@)	0.11	0.30***
(144) Age at NSSI onset (#)	−0.01	−0.18***
(150) *N* of NSSI methods (#)	0.13**	0.41***
(151) NSSI medical attention (@)	0.09	0.06
(153) Automatic negative reinforcement (&)	0.06	0.49***
(154) Automatic positive reinforcement (&)	0.14**	0.50***
(155) Social positive reinforcement (&)	0.42***	0.02
(156) Social negative reinforcement (&)	0.40***	0.18***
**Precipitants**		
(157) Family (&)	0.19***	0.16***
(158) Friends (&)	0.14**	0.19***
(159) Relationships (&)	0.19***	0.15***
(160) Peers (&)	0.05	0.09
(161) Work/school (&)	0.09	0.22***
(162) Mental state (&)	0.06	0.32***
(163) Drugs/alcohol use (% of time) (#)	0.17***	0.14**
(165) No. peers with NSSI before 1st time (#)	0.05	−0.07
(166) No. peers with NSSI after 1st time (#)	0.05	0.03
(167) Peer influence before 1st time? (&)	0.16**	−0.05
(167) Peer influence after 1st time? (&)	0.16**	0.03
Future likelihood of the NSSI (&)	0.00	0.35***

SI, suicidal ideation; SA, suicide attempt; NSSI, nonsuicidal self-injury; BDI, Beck’s depression inventory; STAI-S, state–trait anxiety inventory-sate; STAI-T, state–trait anxiety inventory-trait; PID5BF + M, personality inventory for DSM-5 and ICD-11 brief form plus–modified; SITBI, self-injurious thoughts and behaviors interview. #, Pearson correlation; (#), biserial correlation; (&), polyserial correlation. *p < 0.05, **p < 0.01, ***p < 0.001. @, biserial correlation.

Intrapersonal NSSI functions were significantly positively correlated with 12 months history of NSSI (*r* = 0.30), number of NSSI methods (*r* = 0.41), automatic negative (*r* = 0.49) and positive reinforcement (*r* = 0.50), and likelihood of future NSSI (*r* = 0.35). Interpersonal functions had significant positive correlations with both negative (*r* = 0.40) and positive social reinforcement (*r* = 0.42).

## 4. Discussion

### 4.1. Sample characteristics

As the presence of NSSI and SI was assessed in all patients admitted to the inpatient unit, we were able to determine the prevalence of combined NSSI+SI in the hospital population of patients with non-psychotic mental disorders, which was 18.0%. Basic socio-demographic and clinical characteristics of the sample are shown in Table 1. The predominance of women in the sample is consistent with data on their greater use of psychiatric care. Recent study has found that over 76% of patients in the Moscow clinic for patients with non-psychotic mental disorders are women (55). On the other hand, the predominance of young people, those assigned female at birth and a significant proportion of participants with alternative gender identities is consistent with findings from studies conducted in other countries (56–58).

The distribution of diagnoses in the sample reflects the peculiarities of the organization of psychiatric services in the Russian Federation. In Russia, clinics treat patients with so-called non-psychotic mental disorders separately from patients with primary psychotic disorders, patients with mood disorders with psychotic features and patients with addictive or organic psychotic disorders. At the same time, patients with schizotypal and borderline personality disorders, even if they have transient psychotic symptoms, are predominantly treated in clinics for patients with non-psychotic mental disorders.

### 4.2. Internal consistency and factor structure

The Russian version of the ISAS-II has good overall internal consistency (ω = 0.85), and the omega coefficients for the Intrapersonal (0.83) and Interpersonal (0.85) functions were either greater than 0.80 (Table 3). These data indicate a good internal consistency between these factors and are in line with the results of a study by Klonsky and Glenn (23) (Intrapersonal – 0.88 and Interpersonal – 0.89) and some other studies (61). Interestingly, the internal consistency of the Intrapersonal factor functions was higher (range 0.71–0.83) than that of some of the Interpersonal factor functions (range 0.53–0.78). At the same time, although most of the NSSI motives had acceptable internal consistency (ω ≥ 0.70), the coefficients for “Self-care,” “Sensation seeking,” “Interpersonal boundaries” and “Peer bonding” ranged between 0.55 and 0.70, indicating less adequate reliability. The correlation between the Intrapersonal and Interpersonal functions in our sample was low (0.22). The average inter-item correlations for the Interpersonal and Intrapersonal functions and most of the subfunctions were within the acceptable range of 0.15–0.50.

An analysis of the distribution of items by factor (Intrapersonal and Interpersonal) in the Russian version of the ISAS-II revealed a number of differences from the original version (Table 2). The main difference between the Russian version of the ISAS-II and the original version of the questionnaire was that all 3 items of the “Marking distress” subscale loaded more strongly on the Interpersonal factor. Previously, a study by Reinhardt et al. (62) found that “Marking distress” on a par with “Interpersonal boundaries,” “Sensation Seeking,” “Toughness” and “Autonomy” motives may have both Intrapersonal and Interpersonal components. In the same study, the “Marking distress” function had a salient loading on the Interpersonal factor in male adolescents. Our results are consistent with the study by Vigfusdottir et al. (63), conducted on a sample of Norwegian students, which also showed that the “Marking distress” function loaded more on the Interpersonal factor, in contrast to the original study by Klonsky and Olino (22). In the Korean study (28), − item 11 of the “Marking distress” function also loaded on interpersonal rather than intrapersonal functions.

Another feature of the Russian version of the ISAS-II was that the item 17 (“Creating a physical injury that is easier to care for than my emotional distress”), which in the original version belonged to the Interpersonal function “Self-care” (self-injuring to create a physical wound that one can care for more easily than one’s emotional distress) (23), had a greater load on the Intrapersonal factor. The authors originally expected the “Self-care” function to be theoretically related to the intrapersonal factor, but in the original study this function had a higher load on the Interpersonal (0.41) than on the Intrapersonal factor (0.33) (23). It is interesting to note that in the original study the loading on the Interpersonal factor was only slightly higher than on the Intrapersonal factor. In a more recent study by Klonsky et al. (64), item analysis was performed and showed that item 17 loaded on the Intrapersonal (0.50) rather than the Social factor (0.26), which is fully consistent with our results. In the Russian version of the ISAS-II, the other two items of the “Self-care” function had high loadings on the Interpersonal factor, but the factor loadings were low. The “Self-care” function was also clearly loaded on the Intrapersonal function in the studies by Kortge et al. (65), Vigfusdottir et al. (63), and Pérez et al. (25). In the last of the above-mentioned studies (25), the model in which the “Self-care” function was included in the Intrapersonal factor was found to be preferable to the original model in which it was identified as an Interpersonal function. It is also noteworthy that the other “Self-care” function items, although more heavily loaded on the Interpersonal factor, had some of the lowest loadings of all questionnaire items (0.4 for item 4 and 0.35 for item 30). Given the instability of the “self-care” subfunction (both the items within it and the subfunction as a whole) identified in several studies, we believe that developing a revised version of the ISAS based on the experience of the validations may be the best decision. A new version of the instrument should either clarify the wording of the questions or remove this function altogether.

Another ISAS-II item that showed low factor loadings on both factors was item 7 (“Doing something to generate excitement or exhilaration”), which refers to the “Sensation-seeking” function. Previously, the Korean study (28) found that, contrary to the original research, item 7 loaded more on the Intrapersonal than on the Interpersonal function.

### 4.3. NSSI functions in patients with suicidal ideation

In our study, patients practiced in NSSI for both Intrapersonal and Interpersonal motives (Table 3), which is consistent with most studies, but the prevalence differed from that presented in the meta-analysis by Taylor et al. (66). The three most common Intrapersonal NSSI functions in our sample were “Affect regulation” (96.1%), “Self-Punishment” (87.1%), and “Anti-Suicide” (75.9%), and the most common Interpersonal functions were “Marking distress” (78.8%), “Self-care” (68.9%), and “Interpersonal influence” (54.9%). In a meta-analysis by Taylor et al. (66), avoidance or escape from an unwanted internal state was also the most common Intrapersonal motive for NSSI, while communicating the level of distress and interpersonal influence were found to be the most common Intrapersonal motives. It is noteworthy that the “Self-care” motive for NSSI in the Russian version of the ISAS-II includes both Intrapersonal and Interpersonal items and cannot be completely reduced to just one of the two functions.

### 4.4. Correlations between NSSI functions and personality traits

We conducted a correlational analysis of Interpersonal and Intrapersonal functions with sociodemographic, clinical and personality profile variables. We also examined correlations between ISAS-II functions and parameters from the SITBI Non-suicidal self-injury module (items 144–169). We found small positive correlations (0.3–0.5) between Intrapersonal NSSI functions and lower age, higher BDI score, NSSI episode in the past 12 months, higher number of NSSI methods, “mental state at the time” as a cause of NSSI, and self-reported high likelihood of engaging in NSSI in the future. Our findings in the Russian-speaking patients on the relationship between Intrapersonal functions and the less favorable course of NSSI in patients with non-psychotic mental disorders are consistent with the results of studies conducted in other linguistic and cultural samples (67, 68).

To the best of the authors’ knowledge, no correlational analysis has previously been conducted between DSM-5 personality trait domains and NSSI functions. At the same time, the relationship between NSSI behavior and higher levels of Negative affectivity, Detachment, Antagonism and Psychoticism has been previously reported by researchers (69–73). The Psychoticism domain of the PID-5 includes traits such as Unusual Beliefs & Experiences, Eccentricity, and Perceptual Dysregulation (74). According to Peng et al., patients with depression and NSSI have significantly higher levels of psychoticism than healthy controls and patients with depression alone (73). In addition, regression analysis showed that psychoticism was an independent risk factor for NSSI in depressed patients. These data are supported by the results of the study by Kang et al. (74). In our study, Intrapersonal NSSI functions had the highest correlations with psychoticism (0.32). In our opinion, this may be explained by the fact that psychoticism is associated with a well-known difficulty in dealing effectively with emotional conflict. For example, a study by Granieri et al. found that two immature defense mechanisms – autistic fantasy and isolation – predicted the PID-5 Psychoticism domain in participants (75). While attempts to cope with the unwanted effects of dissociation are among the most common Intrapersonal motives for NSSI, psychoticism has been repeatedly reported to be associated with a high risk for the development of dissociative phenomena (76).

In our study, NSSI Interpersonal functions had the highest correlations with the Antagonism domain (0.25). An association between NSSI and low agreeableness has also been reported in some previous studies (76–78). This variable indirectly reflects antagonism, which, according to the authors of the PID-5 (74), includes traits such as Manipulativeness, Deceitfulness and Grandiosity. The relationship found between Interpersonal functions and Antagonism is consistent with the suggestion by some authors that some NSSI behaviors directed at the environment may involve aggressive goals (e.g., “Revenge” motives).

The SITBI questions related to self-harm motives (items 153–156) had the highest correlations with NSSI functions. The results were as expected: Interpersonal functions correlated with “… to communicate with someone else or to get attention?” (0.42) and “… to get out of doing something or to get away from others?” (0.40), and Intrapersonal functions with statements such as “… a way to get rid of bad feelings?” (0.49) and “… to feel something, because you were feeling numb or empty?” (0.50). These data provide general support for the convergent validity of the SITBI, but it is worth noting that none of the correlations exceeded the level of 0.5.

## 5. Conclusion

The results of our study indicate that the Russian version of the ISAS-II is a valid and reliable instrument for assessing NSSI functions in a population at high risk for suicide attempts. Factor analysis of the Russian version of the ISAS-II revealed a two-factor structure (Intrapersonal and Interpersonal factors) of the instrument. The main difference between the Russian version of the instrument and the original study is that all 3 items of the subfunction “Marking distress” load more heavily on the interpersonal factor. The three most common Intrapersonal NSSI functions in patients with suicidal ideation were “Affect regulation,” “Self-punishment,” and “Anti-Suicide,” and the Interpersonal functions were “Marking distress,” “Self-care,” and “Interpersonal Influence.” In people with non-psychotic mental disorders and suicidal thoughts, Interpersonal functions are associated with more severe depressive symptoms, less favorable course of NSSI and psychoticism personality traits. Validation of the ISAS-II in Russian will help to develop more accurate therapeutic strategies for people with specific motives for NSSI and will be useful for Russian mental health professionals in assessing the further course of NSSI.

## Data availability statement

The raw data supporting the conclusions of this article will be made available by the authors upon request.

## Ethics statement

All procedures in studies involving human participants were conducted in accordance with the ethical standards of the Research Ethics Committee of the Moscow Research and Clinical Center for Neuropsychiatry. Informed consent was obtained from all participants included in the study. The studies were conducted in accordance with the local legislation and institutional requirements. The participants provided their written informed consent to participate in this study.

## Author contributions

MZ: Conceptualization, Funding acquisition, Methodology, Writing – original draft, Writing – review & editing. GK: Data curation, Writing – original draft. SP: Investigation, Writing – original draft. IM: Writing – original draft. NV: Writing – original draft, Investigation. AnG: Formal analysis, Methodology, Writing – original draft. AY: Formal analysis, Software, Writing – original draft. AlG: Project administration, Resources, Writing – review & editing.

## References

[1] Naghavi M. Global, regional, and national burden of suicide mortality 1990 to 2016: systematic analysis for the global burden of disease study 2016. BMJ. (2019) 364:l94. doi: 10.1136/BMJ.L94 31339847 PMC6598639

[2] Hedegaard HCSWM. Suicide mortality in the United States, 1999–2019. NCHS Data Brief. (2021) 398:1–8. doi: 10.15620/cdc:101761 33663651

[3] Santucci C Carioli G Bertuccio P Malvezzi M Pastorino U Boffetta P. Progress in cancer mortality, incidence, and survival: a global overview. Eur J Cancer Prev. (2020) 29:367–81. doi: 10.1097/CEJ.0000000000000594, PMID: 32740162

[4] Bhatnagar P Wickramasinghe K Wilkins E Townsend N. Trends in the epidemiology of cardiovascular disease in the UK. Heart. (2016) 102:1945–52. doi: 10.1136/HEARTJNL-2016-309573, PMID: 27550425 PMC5256396

[5] Hamza CA Stewart SL Willoughby T. Examining the link between nonsuicidal self-injury and suicidal behavior: a review of the literature and an integrated model. Clin Psychol Rev. (2012) 32:482–95. doi: 10.1016/J.CPR.2012.05.003 22717336

[6] Ye Z Xiong F Li W. A meta-analysis of co-occurrence of non-suicidal self-injury and suicide attempt: implications for clinical intervention and future diagnosis. Front Psych. (2022) 13:976217. doi: 10.3389/FPSYT.2022.976217/BIBTEX, PMID: 36032240 PMC9411747

[7] Paul E Tsypes A Eidlitz L Ernhout C Whitlock J. Frequency and functions of non-suicidal self-injury: associations with suicidal thoughts and behaviors. Psychiatry Res. (2015) 225:276–82. doi: 10.1016/J.PSYCHRES.2014.12.026 25592979

[8] Swannell SV Martin GE Page A Hasking P St John NJ. Prevalence of nonsuicidal self-injury in nonclinical samples: systematic review, meta-analysis and meta-regression. Suicide Life Threat Behav. (2014) 44:273–303. doi: 10.1111/SLTB.12070, PMID: 24422986

[9] Wang L Liu J Yang Y Zou H. Prevalence and risk factors for non-suicidal self-injury among patients with depression or bipolar disorder in China. BMC Psychiatry. (2021) 21:389. doi: 10.1186/S12888-021-03392-Y, PMID: 34348675 PMC8335871

[10] Cucchi A Ryan D Konstantakopoulos G Stroumpa S Kaçar AS Renshaw S. Lifetime prevalence of non-suicidal self-injury in patients with eating disorders: a systematic review and meta-analysis. Psychol Med. (2016) 46:1345–58. doi: 10.1017/S0033291716000027, PMID: 26954514

[11] World Health Organization. (2021). Suicide worldwide in 2019: Global health estimates. World Health Organization, Geneva. Licence: CC BY-NC-SA 3.0 IGO. Available at: https://apps.who.int/iris/rest/bitstreams/1350975/retrieve (Accessed June 21, 2023).

[12] Vogelzang BH Scutaru C MacHe S Vitzthum K Quarcoo D Groneberg DA. Depression and suicide publication analysis, using density equalizing mapping and output benchmarking. Indian J Psychol Med. (2011) 33:59–65. doi: 10.4103/0253-7176.85397, PMID: 22021955 PMC3195157

[13] Koposov R Stickley A Ruchkin V. Non-suicidal self-injury among incarcerated adolescents: prevalence, personality, and psychiatric comorbidity. Front Psych. (2021) 12:652004. doi: 10.3389/FPSYT.2021.652004, PMID: 34093271 PMC8170036

[14] Zinchuk M Beghi M Beghi E Bianchi E Avedisova A Yakovlev A. Non-suicidal self-injury in Russian patients with suicidal ideation. Arch Suicide Res. (2022) 26:776–800. doi: 10.1080/13811118.2020.1833801, PMID: 33108991

[15] Iznak AF Iznak EV Damyanovich EV Oleichik IV. Differences of EEG frequency and spatial parameters in depressive female adolescents with suicidal attempts and non-suicidal self-injuries. Clin EEG Neurosci. (2021) 52:406–13. doi: 10.1177/1550059421991685, PMID: 33555208

[16] Faura-Garcia J Orue I Calvete E. Clinical assessment of non-suicidal self-injury: a systematic review of instruments. Clin Psychol Psychother. (2021) 28:739–65. doi: 10.1002/CPP.2537, PMID: 33283952

[17] Evseev VD Peshkovskaya AG Bokhan NA Mandel AI. A screening study of non-suicidal forms self-injurious behavior in persons of draft age. Zh Nevrol Psikhiatr Im S S Korsakova. (2021) 121:54–60. doi: 10.17116/JNEVRO202112108154 34481436

[18] Levkovskaya OB Shevchenko YS Danilova LY Grachev VV. A phenomenological analysis of non-suicidal self-injuries in adolescents. Zh Nevrol Psikhiatr Im S S Korsakova. (2017) 117:10–5. doi: 10.17116/JNEVRO20171177110-15 28805754

[19] Calati R Courtet P. Is psychotherapy effective for reducing suicide attempt and non-suicidal self-injury rates? Meta-analysis and meta-regression of literature data. J Psychiatr Res. (2016) 79:8–20. doi: 10.1016/J.JPSYCHIRES.2016.04.003 27128172

[20] Turner BJ Austin SB Chapman AL. Treating nonsuicidal self-injury: a systematic review of psychological and pharmacological interventions. Can J Psychiatr. (2014) 59:576–85. doi: 10.1177/070674371405901103, PMID: 25565473 PMC4244876

[21] Calvo N García-González S Perez-Galbarro C Regales-Peco C Lugo-Marin J Ramos-Quiroga JA. Psychotherapeutic interventions specifically developed for NSSI in adolescence: a systematic review. Eur Neuropsychopharmacol. (2022) 58:86–98. doi: 10.1016/J.EURONEURO.2022.02.009, PMID: 35325633

[22] Klonsky ED Olino TM. Identifying clinically distinct subgroups of self-injurers among young adults: a latent class analysis. J Consult Clin Psychol. (2008) 76:22–7. doi: 10.1037/0022-006X.76.1.22 18229979

[23] Klonsky ED Glenn CR. Assessing the functions of non-suicidal self-injury: psychometric properties of the inventory of statements about self-injury (ISAS). J Psychopathol Behav Assess. (2009) 31:215–9. doi: 10.1007/S10862-008-9107-Z 29269992 PMC5736316

[24] Kostić J Žikić O Stankovic M Nikolić G. Nonsuicidal self-injury among adolescents in south-East Serbia. Int J Pediatr Adolesc Med. (2019) 6:131–4. doi: 10.1016/J.IJPAM.2019.06.002, PMID: 31890837 PMC6926179

[25] Pérez S García-Alandete J Cañabate M Marco JH. Confirmatory factor analysis of the inventory of statements about self-injury in a Spanish clinical sample. J Clin Psychol. (2020) 76:102–17. doi: 10.1002/JCLP.22844, PMID: 31454078

[26] Reinhardt M Kökönyei G Rice KG Drubina B Urbán R. Functions of nonsuicidal self-injury in a Hungarian community adolescent sample: a psychometric investigation. BMC Psychiatry. (2021) 21:618. doi: 10.1186/S12888-021-03613-4, PMID: 34886827 PMC8662905

[27] Rezaei O Athar ME Ebrahimi A Jazi EA Karimi S Ataie S. Psychometric properties of the persian version of the inventory of statements about self-injury (ISAS). Borderline Personal Disord Emot Dysregul. (2021) 8:27. doi: 10.1186/S40479-021-00168-4, PMID: 34772468 PMC8588687

[28] Kim S Kim Y Hur JW. Nonsuicidal self-injury among Korean young adults: a validation of the Korean version of the inventory of statements about self-injury. Psychiatry Investig. (2019) 16:270–8. doi: 10.30773/PI.2019.01.23, PMID: 30947497 PMC6504776

[29] Bildik T Somer O Kabukçu Başay B Başay Ö Özbaran B. The validity and reliability of the turkish version of the inventory of statements about self-injury. Turk Psikiyatri Derg. (2013) 24:41–9. doi: 10.5080/U6901 23446540

[30] Nisar H Aqeel M Ahmad A. Indigenous need arise to protect human from self-harm behavior in Pakistan: translation and validation of inventory of statements about self-injury. Int J Hum Rights Healthc. (2020) 13:421–33. doi: 10.1108/IJHRH-10-2019-0080

[31] Lindholm T Bjärehed J Lundh LG. Functions of nonsuicidal self-injury among young women in residential care: a pilot study with the Swedish version of the inventory of statements about self-injury. Cogn Behav Ther. (2011) 40:183–9. doi: 10.1080/16506073.2011.565791, PMID: 21877957

[32] Daukantaitė D Lantto R Liljedahl SI Helleman M Westling S. One-year consistency in lifetime frequency estimates and functions of non-suicidal self-injury in a clinical sample. Front Psych. (2020) 11:538. doi: 10.3389/FPSYT.2020.00538, PMID: 32612546 PMC7308529

[33] Reinhardt M Rice KG Horváth Z. Non-suicidal self-injury motivations in the light of self-harm severity indicators and psychopathology in a clinical adolescent sample. Front Psych. (2022) 13:1046576. doi: 10.3389/FPSYT.2022.1046576/BIBTEX, PMID: 36532173 PMC9751932

[34] Khutoryanskaya JV Pozdnyak VV Grechanyy SV. Non-suicidal self-injurious behavior in adolescents. Zh Nevrol Psikhiatr Im S S Korsakova. (2022) 122:105–10. doi: 10.17116/JNEVRO2022122121105 36537640

[35] Zinchuk M Kustov G Pashnin E Gersamia A Rider F Voinova N. Not always that EASI: validating the Russian version of the epilepsy anxiety survey instrument and its brief counterpart. Epilepsy Behav. (2022) 133:108801. doi: 10.1016/J.YEBEH.2022.108801, PMID: 35753109

[36] Scott AJ Sharpe L Thayer Z Miller LA Hunt C MacCann C. Design and validation of two measures to detect anxiety disorders in epilepsy: the epilepsy anxiety survey instrument and its brief counterpart. Epilepsia. (2019) 60:2068–77. doi: 10.1111/EPI.16348, PMID: 31560136

[37] Zarghami M Babakhanian M Asgarabad MH Ghazanfanpour M Akrami FS Nazeri N. Psychometric properties of the inventory of statements about self-injury (ISAS) in Iranian opioid and alcohol abusers. Iran J Psychiatry Behav Sci. (2020) 14. doi: 10.5812/IJPBS.88494

[38] Iijima Y Uemura M Katsuragwa T Shimada H. Development of the Japanese version of the inventory of statements about self-injury and classification of nonsuicidal self-injury in adolescents based on its functions. J Health Psychol Res. (2021) in press 33:103–14. doi: 10.11560/JHPR.200511141

[39] Castro Silva E Benjet C Juárez García F Jurado Cárdenas S Lucio Gómez-Maqueo ME Valencia CA. Adaptación y propiedades psicométricas del Inventory of Statements About Self-injury en estudiantes mexicanos. Acta Investig Psicol. (2016) 6:2544–51. doi: 10.1016/J.AIPPRR.2016.08.004

[40] Nock MK Holmberg EB Photos VI Michel BD. Self-injurious thoughts and Behaviors interview: development, reliability, and validity in an adolescent sample. Psychol Assess. (2007) 19:309–17. doi: 10.1037/1040-3590.19.3.309, PMID: 17845122

[41] Zinchuk M Kustov G Beghi M Voinova N Pashnin E Beghi E. Factors associated with non-binary gender identity in psychiatric inpatients with suicidal ideation assigned female at birth: a case-control study. Arch Sex Behav. (2022) 51:3601–12. doi: 10.1007/S10508-022-02424-2, PMID: 36109451

[42] Markon KE Quilty LC Bagby RM Krueger RF. The development and psychometric properties of an informant-report form of the personality inventory for DSM-5 (PID-5). Assessment. (2013) 20:370–83. doi: 10.1177/1073191113486513, PMID: 23612961

[43] Kustov GV Zinchuk MS Pashnin EV Voinova NI Popova SB Gersamia AG. Psychometric properties of the Russian version of the PID-5-BF questionnaire. Psychol J High Sch Econ. (2022) 19:521–42. doi: 10.17323/1813-8918-2022-3-521-542

[44] Bach B Kerber A Aluja A Bastiaens T Keeley JW Claes L. International assessment of DSM-5 and ICD-11 personality disorder traits: toward a common nosology in DSM-5.1. Psychopathology. (2020) 53:179–88. doi: 10.1159/000507589 32369820

[45] Zinchuk M Kustov G Bach B Pashnin E Gersamija A Yakovlev A. Evaluation of a 36-item measure of ICD-11 and DSM-5 personality disorder trait domains and facets in Russian inpatients. Psychol Assess. (2023) 35:e22–30. doi: 10.1037/PAS0001223, PMID: 36931820

[46] Beck AT Ward CH Mendelson M Mock J Erbaugh J. An inventory for measuring depression. Arch Gen Psychiatry. (1961) 4:561–71. doi: 10.1001/ARCHPSYC.1961.01710120031004 13688369

[47] Tarabrina NV. Praktikum po psikhologii postravmaticheskogo stressa. St. Petrersburg: Piter Publ (2001).

[48] Spielberger Cd. (1983). Manual for the state-trait anxiety inventory (stai). CA: Consulting Psychologists.

[49] Juri K. Kratkoye rukovodstvo k primeneniyu shkaly reaktivnoy i lichnostnoy trevozhnosti ChD Spilbergera. Leningrad: LNIITEK (1976). 40 p.

[50] Evgenii V Elena N Julia B. On the question of the psychometric reliability of some psychological techniques. Theor Exp Psychol. (2019) 12:61–8.

[51] Mplus users guide v6. – Muthén & Muthén. Available at: https://www.yumpu.com/en/document/read/40845405/mplus-users-guide-v6-muthen-muthen (Accessed June 21, 2023).

[52] Olaru G Witthöft M Wilhelm O. Methods matter: testing competing models for designing short-scale big-five assessments. J Res Pers. (2015) 59:56–68. doi: 10.1016/J.JRP.2015.09.001

[53] McDonald RP. Test theory: a unified treatment. (1999) 485. Available at: https://books.google.com/books/about/Test_Theory.html?hl=ru&id=acww1KB6BdEC (Accessed June 23, 2023).

[54] Paulsen J BrckaLorenz A. Internal consistency. (2017) Available at: https://scholarworks.iu.edu/dspace/handle/2022/24498 (Accessed June 23, 2023).

[55] Zinchuk M Beghi M Castelpietra G Ferrari S Pashnin E Guekht A. The impact of COVID-19 quarantine on mental health: an observational study from an outpatient service for non-psychotic patients in Russia (Moscow). Emerg Care J. (2023) 19. doi: 10.4081/ecj.2023.10994

[56] Surace T Fusar-Poli L Vozza L Cavone V Arcidiacono C Mammano R. Lifetime prevalence of suicidal ideation and suicidal behaviors in gender non-conforming youths: a meta-analysis. Eur Child Adolesc Psychiatry. (2021) 30:1147–61. doi: 10.1007/S00787-020-01508-5, PMID: 32170434

[57] Bresin K Schoenleber M. Gender differences in the prevalence of nonsuicidal self-injury: a meta-analysis. Clin Psychol Rev. (2015) 38:55–64. doi: 10.1016/J.CPR.2015.02.009 25795294

[58] Wilkinson PO Qiu T Jesmont C Neufeld SAS Kaur SP Jones PB. Age and gender effects on non-suicidal self-injury, and their interplay with psychological distress. J Affect Disord. (2022) 306:240–5. doi: 10.1016/J.JAD.2022.03.021 35304237

[59] OECD. Education at a Glance 2019. Education at a glance: OECD indicators (2019). OECD Publishing.

[60] Brief results of the sample survey “family and fertility”. (2009). Available at: https://rosstat.gov.ru/storage/mediabank/%D0%9A%D1%80%D0%B0%D1%82%D0%BA%D0%B8%D0%B5%20%D0%B8%D1%82%D0%BE%D0%B3%D0%B8%20%D0%B2%D1%8B%D0%B1%D0%BE%D1%80%D0%BE%D1%87%D0%BD%D0%BE%D0%B3%D0%BE%20%D0%BE%D0%B1%D1%81%D0%BB%D0%B5%D0%B4%D0%BE%D0%B2%D0%B0%D0%BD%D0%B8%D1%8F%20_%D0%A1%D0%B5%D0%BC%D1%8C%D1%8F%20%D0%B8%20%D1%80%D0%BE%D0%B6%D0%B4%D0%B0%D0%B5%D0%BC%D0%BE%D1%81%D1%82%D1%8C_.html (Accessed October 23, 2023).

[61] De Salve F Placenti C Tagliabue S Rossi C Malvini L Percudani M. Are PID-5 personality traits and self-harm attitudes related? A study on a young adult sample pre-post COVID-19 pandemic. J Affect Disord Rep. (2023) 11:100475. doi: 10.1016/J.JADR.2023.100475, PMID: 36620760 PMC9811916

[62] Reinhardt M Kökönyei G Drubina B Urbán R. Gender differences in the psychometric characteristics of the inventory of statements about self-injury in a community adolescent sample. BMC Psychiatry (2021) 21:618. doi: 10.21203/RS.3.RS-151042/V1 34886827 PMC8662905

[63] Vigfusdottir J Dale KY Gratz KL Klonsky ED Jonsbu E Høidal R. The psychometric properties and clinical utility of the Norwegian versions of the deliberate self-harm inventory and the inventory of statements about self-injury. Curr Psychol. (2022) 41:6766–76. doi: 10.1007/S12144-020-01189-Y

[64] Klonsky ED Glenn CR Styer DM Olino TM Washburn JJ. The functions of nonsuicidal self-injury: converging evidence for a two-factor structure. Child Adolesc Psychiatry Ment Health. (2015) 9:44. doi: 10.1186/S13034-015-0073-4, PMID: 26421059 PMC4586000

[65] Kortge R Meade T Tennant A. Interpersonal and intrapersonal functions of deliberate self-harm (DSH): a psychometric examination of the inventory of statements about self-injury (ISAS) scale. Behav Chang. (2013) 30:24–35. doi: 10.1017/BEC.2013.3

[66] Taylor PJ Jomar K Dhingra K Forrester R Shahmalak U Dickson JM. A meta-analysis of the prevalence of different functions of non-suicidal self-injury. J Affect Disord. (2018) 227:759–69. doi: 10.1016/J.JAD.2017.11.073, PMID: 29689691

[67] Muehlenkamp J Brausch A Quigley K Whitlock J. Interpersonal features and functions of nonsuicidal self-injury. Suicide Life Threat Behav. (2013) 43:67–80. doi: 10.1111/J.1943-278X.2012.00128.X, PMID: 23082783

[68] Turner BJ Chapman AL Layden BK. Intrapersonal and interpersonal functions of non suicidal self-injury: associations with emotional and social functioning. Suicide Life Threat Behav. (2012) 42:36–55. doi: 10.1111/J.1943-278X.2011.00069.X, PMID: 22276747

[69] Canol T Sapmaz SY Barut EA Cakir ADU Bilac O Kandemir H. Nonsuicidal self-injury in adolescents: role of sociodemographic and clinical factors, emotion regulation, and maladaptive personality traits. Dusunen Adam J Psychiatry Neurol Sci. (2022) 35:155–64. doi: 10.14744/DAJPNS.2022.00188

[70] Davis KC Anderson JL. Psychological pain: a moderating factor between personality psychopathology and self-harm. J Am Coll Heal. (2021) 71:1436–44. doi: 10.1080/07448481.2021.1928677 34133265

[71] Su-gai L Jing Y. Personality Traits of Chinese Adolescents with Non-suicidal Self Injury and Suicide Attempt. Sichuan Da Xue Xue Bao Yi Xue Ban. (2014) 45:970–973. 25571726

[72] Krueger RF Derringer J Markon KE Watson D Skodol AE. Initial construction of a maladaptive personality trait model and inventory for DSM-5. Psychol Med. (2012) 42:1879–90. doi: 10.1017/S0033291711002674, PMID: 22153017 PMC3413381

[73] Peng B Liao J Li Y Jia G Yang J Wu Z. Personality characteristics, defense styles, borderline symptoms, and non-suicidal self-injury in first-episode major depressive disorder. Front Psychol. (2023) 14:989711. doi: 10.3389/FPSYG.2023.989711, PMID: 36777206 PMC9909038

[74] Kang L Li R Liu H Ma S Sun S Zhang N. Nonsuicidal self-injury in undergraduate students with major depressive disorder: the role of psychosocial factors. J Affect Disord. (2021) 290:102–8. doi: 10.1016/J.JAD.2021.04.083, PMID: 33993076

[75] Granieri A La Marca L Mannino G Giunta S Guglielmucci F Schimmenti A. The relationship between Defense patterns and DSM-5 maladaptive personality domains. Front Psychol. (2017) 8:1926. doi: 10.3389/FPSYG.2017.01926, PMID: 29163301 PMC5673655

[76] Kiekens G Bruffaerts R Nock MK Van de Ven M Witteman C Mortier P. Non-suicidal self-injury among Dutch and Belgian adolescents: personality, stress and coping. Eur Psychiatry. (2015) 30:743–9. doi: 10.1016/J.EURPSY.2015.06.007, PMID: 26260264

[77] Brown SA. Personality and non-suicidal deliberate self-harm: trait differences among a non-clinical population. Psychiatry Res. (2009) 169:28–32. doi: 10.1016/J.PSYCHRES.2008.06.005, PMID: 19616308

[78] MacLaren VV Best LA. Nonsuicidal self-injury, potentially addictive behaviors, and the five factor model in undergraduates. Pers Individ Dif. (2010) 49:521–5. doi: 10.1016/J.PAID.2010.05.019

