# Supplementary material for: Functions of nonsuicidal self-injurious behavior in Russian patients with suicidal ideation
Source: Front Public Health. 2023 Nov 7;11:1270944. doi: 10.3389/fpubh.2023.1270944 (PMC10660280; doi:10.3389/fpubh.2023.1270944)
Supplement: Supplementary file 1 [file Data_Sheet_1.PDF]

Опросник утверждений о самоповреждении (ОУОС)

Раздел I. Поведение

Этот опросник касается особенностей самоповреждающего поведения.

Пожалуйста, выберите те действия, которые Вы совершали намеренно и без суицидальных целей (т. е. НЕ для того, чтобы уйти из жизни).

1. Пожалуйста, укажите для каждого из нижеперечисленных вариантов намеренного несуицидального самоповреждения то количество раз, которое Вы совершили его на протяжении жизни (например, 0, 10, 100, 500)

Нанесение порезов______	Наносили сильные царапины____
Нанесение укусов_______	Били себя или ударялись о стены или предметы________
Нанесение ожогов_______	Мешали зажить ранам (например, отдирая корку)_____
Вырезание на коже букв, слов, цифр или знаков________	Тёрли кожу о грубые поверхности___
Щипали себя_______	Втыкали иглы в тело___________
Дёргали себя за волосы_____	Принимали внутрь опасные вещества _____
Другие __________________, ____	

****

Важно: **Если Вы совершали одно или более из действий перечисленных выше – заполните оставшуюся часть опросника. Если Вы не совершали ни одного из вышеперечисленных действий – Вы завершили работу с данным опросником.**

****

2. Если какое-то из самоповреждений является для Вас *основным*, пожалуйста, обведите его на первой странице.

3. В каком возрасте Вы:

Впервые нанесли себе самоповреждение? _______

Последний раз наносили себе самоповреждение__________
(Приблизительно – день/месяц/год)

4. Испытываете ли Вы физическую боль при нанесении самоповреждения?

Пожалуйста, выберите: ДА ИНОГДА НЕТ

5. В момент нанесения себе повреждений Вы находитесь один (одна)?

Пожалуйста, выберите: ДА ИНОГДА НЕТ

6. Сколько обычно проходит времени с момента когда у Вас появилось желание нанести самоповреждение до того как Вы это сделаете?

Пожалуйста, обведите наиболее подходящий вариант:

< 1 часа	1 - 3 часа	3 - 6 часов
6 - 12 часов	12 - 24 часа	> 1 дня

7. Вы хотите (хотели в прошлом) перестать наносить себе повреждения?

Пожалуйста, обведите ответ ДА НЕТ

Раздел II. Функции

Инструкции

Этот опросник был создан, чтобы помочь нам лучше понять опыт людей, прибегающих к несуицидальным самоповреждениям. Ниже приведён список утверждений, которые могут соответствовать или не соответствовать Вашим переживаниям при самоповреждении. Пожалуйста, отметьте рядом с каждым из утверждений подходящий Вам вариант

- Обведите **0** если утверждение **совершенно не совпадает** с Вашими переживаниями
- Обведите **1** если утверждение **частично совпадает** с Вашими переживаниями
- Обведите **2** если утверждение **полностью совпадает** с Вашими переживаниями

**Когда я наношу себе повреждения, я …**	Ответ		
1. … успокаиваю себя	0	1	2
2. … провожу границу между собой и окружающими	0	1	2
3. … наказываю себя	0	1	2
4. … создаю возможность позаботиться о себе (ухаживая за раной)	0	1	2
5. … вызываю боль, чтобы выйти из оцепенения (бесчувствия)	0	1	2
6. … избегаю импульса совершить самоубийство	0	1	2
7. … стараюсь вызвать возбуждение или воодушевление	0	1	2
8. … стараюсь сблизиться со сверстниками	0	1	2
9. … даю другим понять насколько мне эмоционально больно	0	1	2
10. … проверяю, могу ли я вынести боль	0	1	2
11. … создаю физический знак того, как ужасно я себя чувствую	0	1	2
12. … стараюсь в ответ насолить кому-то	0	1	2
13. … доказываю себе, что самодостаточный (ни в ком не нуждаюсь)	0	1	2
14. … сбрасываю накопившееся эмоциональное напряжение	0	1	2
15. … демонстрирую свою обособленность от других людей	0	1	2
16. … выражаю свой гнев на себя за свою ничтожность и бестолковость	0	1	2

Варианты ответа: 0 – совершенно не верно, 1 – частично верно, 2 – полностью верно

17. … создаю физическое повреждение, которое проще залечивать, чем эмоциональные раны	0	1	2
18. … пытаюсь почувствовать хоть что-нибудь (в противоположность бесчувствию), даже если это физическая боль	0	1	2
19. … реагирую на мысли о нежелании жить, при этом, не совершая реальной суицидальной попытки	0	1	2
20. … развлекаю себя или окружающих, делая что-нибудь экстремальное	0	1	2
21. … стараюсь соответствовать окружающим меня людям	0	1	2
22. … ищу заботы или помощи от окружающих	0	1	2
23. … демонстрирую свою твёрдость и силу	0	1	2
24. … доказываю себе, что моя эмоциональная боль реальна	0	1	2
25. … пытаюсь отомстить другим	0	1	2
26. … демонстрирую, что не нуждаюсь в помощи окружающих	0	1	2
27. … уменьшаю тревогу, разочарование, гнев или другие давящие эмоции	0	1	2
28. … ставлю барьер между собой и окружающими	0	1	2
29. … реагирую на чувство недовольства собой или отвращение к себе	0	1	2
30. … даю себе возможность сосредоточиться на приносящим удовлетворение лечении раны	0	1	2
31. … убеждаюсь, что я всё ещё жив, когда не чувствую себя реальным	0	1	2
32. … останавливаю мысли о самоубийстве	0	1	2
33. … проверяю пределы своих возможностей, на манер прыжков с парашютом или других экстремальных занятий	0	1	2
34. … создаю свидетельство дружбы или знак родства с друзьями или любимым	0	1	2
35. … удерживаю любимого человека от ухода или оставления меня	0	1	2
36. … доказываю, что могу вынести физическую боль	0	1	2
37. … делаю знак, что испытываю эмоциональное страдание	0	1	2
38. … пытаюсь сделать больно кому-то из близких	0	1	2
39. … доказываю, что я автономен / независим	0	1	2

Варианты ответа: 0 – совершенно не верно, 1 – частично верно, 2 – полностью верно

(По желанию) Впишите ниже утверждения, которые лучше опишут Ваши переживания, чем перечисленные выше:

(По желанию) Впишите ниже утверждения, которые, по Вашему мнению, следует внести в список, даже если они не касаются именно Вас:
